# Mental health Project ECHO Autism: Increasing access to community mental health services for autistic individuals

**DOI:** 10.1177/13623613211028000

**Published:** 2021-07-03

**Authors:** Nicole Ginn Dreiling, Michal L Cook, Elena Lamarche, Laura Grofer Klinger

**Affiliations:** University of North Carolina at Chapel Hill, USA

**Keywords:** autism, mental health, professional development, Project ECHO, teleconsultation

## Abstract

Despite the high prevalence of co-occurring autism spectrum disorders and mental health condition(s), there exist substantial barriers to mental health treatment for autistic individuals. These barriers are exacerbated by a lack of mental health provider training and self-efficacy in providing adapted services to autistic individuals. One method which has been effective in mitigating similar service gaps is the Extension for Community Healthcare Outcomes (Project ECHO) Autism model, a tele-mentoring platform that connects primary care physicians to autism spectrum disorder experts to improve physicians’ knowledge, self-efficacy, and practice. This study developed and implemented a pilot mental health version of Project ECHO Autism designed to increase mental health provider knowledge, self-efficacy, and problem-solving. Community mental health providers (*N* = 51) participated in the 6-month Project ECHO Autism including mental health–focused didactics and provider case presentations. Analysis of pre- and post-measures revealed improvements in all domains, including significant increases in provider knowledge of autism spectrum disorders, self-efficacy, and problem-solving. Participants additionally reported high satisfaction with their experience. Taken together, preliminary results indicate that Project ECHO Autism may be a feasible, accessible, and effective method for increasing mental health provider competence and ultimately increasing access to services for autistic individuals who have co-occurring mental health diagnoses.

**Lay abstract:**

Although many autistic individuals have additional mental health conditions, most have a hard time getting services from mental health providers. One reason why these services can be hard to access is that many mental health providers do not feel confident in their ability to provide services to autistic individuals. To share autism expertise with local community providers and boost their confidence in working with autistic individuals, we created a mental health version of the Extension for Community Healthcare Outcomes (Project ECHO) Autism virtual teleconsultation program. In this pilot study, we recruited 51 community mental health providers to participate in Project ECHO Autism. During each biweekly session, providers received information from autism experts about how to tailor mental health interventions (e.g. attention-deficit hyperactivity disorder or anxiety interventions) for use with autistic individuals. They also had the opportunity to ask questions and get advice on their current cases. At the end of the 6-month study, mental health providers showed improvements in their confidence, in their knowledge of autism, and in their problem-solving skills. Nearly half (45%) of these providers participated from rural counties, suggesting that the Project ECHO Autism teleconsultation model was able to reach mental health providers who might not have been able to get training otherwise. This study supports the feasibility of using Project ECHO Autism to share autism knowledge with mental health providers, consequently expanding mental health service options for autistic individuals with co-occurring mental health conditions.

The growing prevalence of autism spectrum disorder (ASD) from 1 in 150 in 2002 to 1 in 54 in 2016 represents a 178% increase in school-aged children seeking services, consequently creating a parallel demand for access to necessary treatment (Maenner et al., 2020). The gap between demand and availability of services is an especially significant barrier for minority children and those living in rural communities, both of whom are consistently diagnosed later in life and receive less treatment (Antezana et al., 2017; Maenner et al., 2020; Smith et al., 2020). While autistic children and their families face numerous hurdles to service acquisition, those with co-occurring mental health condition(s) often fall through the cracks altogether (Maddox & Gaus, 2019). Recent meta-analyses of psychiatric comorbidities in autism found that co-occurring mental health conditions such as attention-deficit hyperactivity, anxiety, depression, schizophrenia, obsessive-compulsive, and sleep-wake disorders were all more prevalent in autistic individuals than in the general population (Hossain et al., 2020; Lai et al., 2019; Lugo-Marín et al., 2019). Studies that have measured the overall prevalence of ASD and at least one other co-occurring psychiatric condition have reported a range of 70% (Simonoff et al., 2008) to 92% (Brookman-Frazee et al., 2018). This co-occurrence has been documented consistently from preschool (Muratori et al., 2019) to young adulthood (Jackson et al., 2018) and has also been shown to compound risk for poorer health outcomes (Ahmedani & Hock, 2012) and lower reported quality of life (Mason et al., 2019).

Despite the clear need to treat mental health conditions in autistic individuals, parents of children with diagnoses of both ASD and a psychiatric comorbidity frequently report unmet service needs (Zablotsky et al., 2015). In a sample of 462 parents, 32% reported their child had experienced a mental health crisis in the last month, 25% of whom did not receive any kind of mental health treatment (Vasa et al., 2019).

Although gold standard mental health treatments like Cognitive Behavioral Therapy (CBT) have consistently demonstrated high efficacy in treating anxiety in autistic individuals (Steinbrenner et al., 2020; Walters et al., 2016), community mental health providers receive little training to successfully implement treatment tailored to their needs (Brookman-Frazee, Drahota, et al., 2012; Williams & Haranin, 2016) and consequently often refer out to specialty clinics with extensive waitlists (Maddox & Gaus, 2019). Results from recent parent focus groups reveal that some families wait as long as 6 months to 2 years to get into specialized therapeutic services for their autistic children (Smith-Young et al., 2020). Furthermore, some autistic adolescents and young adults have reported that even after waiting for services, treatment never materialized (Crane et al., 2018). Although community mental health providers have the capacity to adapt and implement treatment to the needs of autistic individuals, there are significant barriers currently inhibiting this treatment avenue for affected families.

## Barriers to treating co-occurring ASD and mental health condition(s)

Two specific problems that perpetuate this gap in service delivery include (1) a disconnect in care coordination and (2) a lack of mental health provider self-efficacy and training (Crane et al., 2018; Maddox & Gaus, 2019). Inclusion criteria for services often restrict eligibility based on either a developmental disability for community mental health clinics or a mental health condition for intellectual and developmental disability specialty clinics, resulting in a referral out when either service system encounters comorbidity (Lake et al., 2014). Mental health providers indicate that they are not trained to provide services for individuals with developmental disabilities including autism, whereas autism providers indicate that they are not trained to provide mental health services (Maddox & Gaus, 2019). As both systems punt referrals to one another, autistic individuals with co-occurring mental health condition(s) go untreated (Crane et al., 2018; Lake et al., 2014; Maddox & Gaus, 2019). While many mental health providers have the skills to deliver adapted care, their lack of training and organizational support to do so precludes the opportunity.

Mental health providers’ low self-confidence and efficacy to work with autistic clients is a significant limitation to service availability; clinicians report more favorable attitudes, greater normative pressure, and higher self-efficacy to provide CBT to non-autistic clients (Maddox, Crabbe, Fishman, et al., 2019). In their survey of 100 community mental health therapists, Brookman-Frazee and colleagues found that although 76% of providers reported having seen an autistic child, therapists perceived working with autistic children as a significant challenge for which they had not been trained (Brookman-Frazee, Drahota, et al., 2012). Similarly, parents of autistic children report parallel barriers to accessing mental health services, including perceived lack of provider knowledge, experience, competence, and confidence, in addition to frustrations with communication breakdown between community mental health and developmental disability service systems (Maddox, Crabbe, Beidas et al., 2019). Furthermore, both autistic adolescents and adults report similar frustrations in accessing mental health services and in the lack of provider understanding of autism (Au-Yeng et al., 2019; Camm-Crosbie et al., 2019; Crane et al., 2018). Autistic adults reported that this absence of understanding increased their feelings of disempowerment, hopelessness, and suicidal ideation (Camm-Crosbie et al., 2019). In a recent report of stakeholder experiences, 69% of autistic adults listed the efficacy of psychological interventions, including CBT, in their desired priorities for autism research, with 89% ranking anxiety treatment as an outcome that mattered the most to them (Benevides et al., 2020).

Significant challenges in the provision of mental health treatment are exacerbated by a lack of autism-specific training (Maddox, Crabbe, Beidas et al., 2019). Importantly, despite their reported barriers to working with this population, many mental health providers have also demonstrated substantial motivation to learn more (Brookman-Frazee, Drahota et al., 2012) and show increased confidence because of ASD-specific training (Williams & Haranin, 2016). In a survey of 50 mental health providers’ use of adapted CBT with autistic clients, providers’ low confidence in working with this population was not associated with years of practice, but rather was positively associated with prior training experience (Cooper et al., 2018). These encouraging results suggest that many providers are interested in engaging in ASD-specific training to increase their self-efficacy.

## Disseminating ASD expertise to community mental health providers

To increase opportunities for autistic individuals to receive evidence-based mental health treatment, there is a fundamental need to empower community providers to include this type of service within their scope of practice. Various studies evaluating ASD-specific training for community mental health providers have yielded significant improvement in patient outcomes and provider confidence (Brookman-Frazee, Drahota, et al., 2012; Brookman-Frazee et al., 2019; Bryson & Ostmeyer, 2014) as well as gains in parental satisfaction and perceived therapeutic alliance with their child’s therapist (Stadnick et al., 2013).

Brookman-Frazee and colleagues’ An Individualized Mental Health Intervention for Children with ASD (AIM HI) (Brookman-Frazee & Drahota, 2010) is one exemplary ASD training model for mental health providers in which participants receive comprehensive in-person introductory education and ongoing weekly group case consultation. Results supported improvement in both provider knowledge and client gains (Brookman-Frazee, Drahota et al., 2012; Brookman-Frazee et al., 2019). Bryson and Ostmeyer’s (2014) dissemination of social skills training for ASD to community mental health providers is another in-person training model that demonstrated increased knowledge for participating providers. In their study, participants received a comprehensive and individualized training experience specific to each participating community mental health site. These studies highlight the feasibility and efficacy of training community mental health providers in evidence-based interventions for ASD. These comprehensive, individualized professional development opportunities, while effective, require extensive time and manpower and are not often available for providers in rural areas with limited access to expertise. Challenges including cost of travel and geographic isolation have been identified as key barriers to training opportunities for rural allied health providers and highlight the potential for technology-enabled distance learning (Berndt et al., 2017). The need persists for innovative models to train community mental health providers to truly build capacity for these evidence-based interventions for specialist populations, particularly in rural or underserved communities.

### Project ECHO Autism

One professional development model that has demonstrated success in disseminating specialty expertise into community settings is the Extension for Community Healthcare Outcomes (Project ECHO) program. Project ECHO was designed as a tele-mentoring platform to virtually connect community-based primary care physicians with specialty experts for didactic training and case consultations (Arora et al., 2010; Mazurek, Harkins, et al., 2020). In this model, local primary care providers (“spokes”) participate in collaborative, guided practice by joining bimonthly videoconference calls with a team of experts (“hub”). During the program, participants are encouraged to consult and support each other to build a provider community of support in addition to receiving expert support through each session. Project ECHO has been used successfully to train primary care providers in best practice treatment for the identification, diagnosis, and management of a variety of medical conditions including autism (Sohl et al., 2017). Providers have consistently reported high satisfaction with the program and demonstrated significant gains in self-efficacy and knowledge of ASD (Mazurek et al., 2017; Mazurek, Curran, et al., 2019; Mazurek, Stobbe, et al., 2019; Mazurek, Parker, et al., 2020).

While Project ECHO has demonstrated great success for medical providers in increasing screening and referral rates, the need to adapt the model to support community mental health providers has been recently recognized. In their recent dissemination of Project ECHO Autism to community-based psychologists, Nowell and colleagues (2020) similarly observed significant gains in provider self-efficacy in screening and diagnostic testing for ASD. The need to adapt the model to support community providers providing mental health treatment to autistic individuals, however, remains. Taken together, improvements in provider self-efficacy and knowledge in ASD screening and diagnosis suggest that Project ECHO Autism could be an effective dissemination tool for providing training specific to mental health treatment.

## Current study

The purpose of this study was to develop and implement a Project ECHO Autism curriculum specifically for community mental health providers. In launching this pilot project, we sought to evaluate the feasibility of using Project ECHO to provide (1) psychoeducation about autism symptoms and co-occurring mental health diagnoses and (2) strategies for implementing evidence-based interventions (e.g. CBT) tailored to meet the needs of autistic clients. We aimed to increase mental health providers’ self-efficacy, knowledge of ASD, and problem-solving skills to ultimately expand the availability of appropriately adapted evidence-based treatment options for autistic individuals with co-occurring mental health conditions.

## Methods

### Sample

Mental health providers in North Carolina signed up to participate in the Project ECHO Autism Mental Health program (“ECHO Autism”). Participants were recruited across four cohorts, each spanning 6 months. Recruitment occurred through listservs of providers who attended previous sponsored educational workshops through the North Carolina (NC) Area Health Education Center (AHEC) program, a statewide network designed to support healthcare professional development activities for rural or under-resourced communities in NC. Specifically, providers were recruited from 27 counties, 16 of which were rural (45.1% of the sample). To allow for supportive, interactive sessions with opportunities for all providers to participate in case-based learning, each cohort was capped at 25 with waitlists created for future ECHO cohorts. In addition to their participation in the ECHO Autism program, each provider was given the option to participate in this research study by completing pre- and post-assessment measures. Initially, across the four cohorts, 86 providers consented to research and completed pre-assessment measures. Because providers typically had full caseloads during their participation in Project ECHO, we set a threshold of 60% attendance to assess for change in realistic and replicable parameters. Providers who attended less than 60% of ECHO sessions (*n* = 4), who attended more than 60% of sessions but failed to complete post-assessment measures (*n* = 1), or who failed to meet both attendance and pre-post questionnaire completion (*n* = 30) were excluded from the analysis, yielding a final sample size of 51 (cohort 1 *n* = 9, cohort 2 *n* = 15, cohort 3 *n* = 17, cohort 4 *n* = 10). Although research eligibility required a minimum of 60% attendance, the majority (88.2%, *n* = 45) of providers included in this study attended at least 80% of ECHO sessions (average attendance per provider was 8.78/10 sessions). Providers were on average 42 years old, predominantly female (94.1%), non-Hispanic or Latinx (98), and White or Caucasian (86.3%). Providers had been in practice for 11 years on average, and the most prevalent specialty was social work (37.3%). See Table 1 for complete demographic information. All providers received continuing education credit through AHEC for the hours they attended.

**Table 1.** Provider and practice characteristics (*N* = 51).

	*M* (*SD*)	Range
Age	42.22 (10.6)	25–66
Years of practice	11.24 (9.9)	1–43
Number of children seen each year	112.7 (146.6)	0–611
Number of autistic children seen each year	20.40 (32.5)	0–150
	*n*	%
Gender		
Female	48	94.1
Ethnicity		
Non-Hispanic or Latinx	50	98.0
Race		
White/Caucasian	44	86.3
Black or African American	6	11.8
American Indian or Alaska Native	1	2.0
Practice setting		
Solo practice	12	23.5
Private group practice	14	27.5
Academic medical center	1	2.0
School	5	9.8
Integrated primary care	5	9.8
State early intervention program	1	2.0
Other	12	23.5
Specialty		
Social worker	19	37.3
Psychologist	14	27.5
Marriage and family therapist	3	5.9
Licensed Clinical Mental Health Counselor	11	21.6
Other	4	7.8
IDD/MH	1	2.0
Early childhood mental health	1	2.0
Services coordinator	1	2.0
Unspecified	1	2.0
Prior ASD training		
Yes	25	49.0
No	26	51.0
If yes, type(s) of training		
Graduate-level course	18	35.3
Workshop/Seminar	38	74.6
Presentation (webinar/conference)	28	54.9
Rotation during internship, fellowship	8	15.7

IDD/MH: Intellectual and Developmental Disability/Mental Health; ASD: autism spectrum disorder.

### ECHO Autism mental health model

This ECHO Autism pilot study was approved by the Institutional Review Board and the Office of Human Research Ethics and consisted of 10 biweekly teleconsultation sessions over 6 months. The pilot followed the Project ECHO model (Arora et al., 2010) of using tele-mentoring to create a “knowledge network” that allowed participants to continue to work with their clients, without having to refer out to specialty clinics, while developing their knowledge through collaborative learning. Each 90-min ECHO Autism meeting included a brief evidence-based didactic followed by case presentations from participating providers. The multidisciplinary expert panel (“hub team”) who facilitated this ECHO Autism program were recruited from a university-based autism outpatient clinic and a regional autism society agency. Specifically, team members included two senior psychologists (one university clinical faculty and one community provider, each with more than 10 years of treatment and evaluation experience with autistic individuals across the lifespan), one parent advocate/professional autism resource specialist, and one university outpatient clinician with a background in education and more than 20 years of clinic-based experience working with autistic individuals. In each cohort, the parent advocate was recruited from the cohort’s local region to provide information about local resources.

The didactic portion of each ECHO Autism clinic presented training to help therapists (1) recognize and understand symptoms of autism and (2) utilize evidence-based strategies to address mental health issues with this population (e.g. how to tailor or adapt CBT for autistic individuals.) The strategies presented in the didactics were chosen from the National Clearinghouse on Autism Evidence and Practice (NCAEP) report on evidence-based practices (Steinbrenner et al., 2020) and included strategies such as visual supports, video modeling, and social narratives that are supported by the current intervention literature (e.g. see Reaven et al., 2018, for a discussion of how to provide community training for mental health clinicians to tailor CBT for autistic clients).

The specific didactic topics were chosen based on feedback from a series of focus groups with rural families who provided input on the mental health needs of their autistic child. The selection of didactic topics was also guided by survey input from mental health professionals about their perceived practice needs when working with autistic clients with co-occurring mental health diagnoses. A full list of didactic topics can be found in Table 2.

**Table 2.** Project ECHO Autism didactic topics.

Session	Didactic Topic
1	Understanding Autism Symptoms and Learning Challenges
2	Introduction to Evidence-Based Intervention Strategies in Autism
3	Providing Parent Support
4	Behavior Management: Evidence-Based Strategies to Tailor Treatment
5	Emotion Regulation Intervention Strategies: Evidence-Based Strategies to Tailor Treatment
6	ASD and Anxiety: Evidence-Based Strategies to Tailor Treatment
7	ASD and ADHD: Evidence-Based Strategies to Tailor Treatment
8	Supporting Families and Clients in the Education Process
9	Evidence-Based Strategies to Build Social Competence
10	*Participant Choice* (e.g. communication strategies, sexuality, and transition to adulthood)

ECHO: Extension for Community Healthcare Outcomes; ASD: autism spectrum disorder; ADHD: attention-deficit hyperactivity disorder.

Case presentations were submitted by participants focused on three primary topic areas: suspected symptoms of ASD and questions of when to refer for evaluation (31.8%), treatment of a variety of mental health challenges (e.g. difficulties with emotion regulation and transition, sleep disturbance, anger and hyperactivity, poor social skills, and depression, 54.5%), and developmental issues (e.g. language development, limited diet, puberty, and transition to adulthood, 11.4%). Although most case presentations were focused on autistic children and adolescents, several participating providers served individuals across the lifespan, and 13.6% of case presentations were adult-focused. During each ECHO Autism session, a summary of the de-identified case was first shared by the presenter, followed by recommendations from the other participants and expert team. A summary of recommendations was later emailed out to all participants, and presenters were invited to present their case for follow-up feedback 4–6 weeks after their original presentation.

Change in provider self-efficacy, ASD knowledge, and problem-solving was observed via pre- and post-test completion of various measures, including a combination of original and adapted surveys from Mazurek and colleagues’ previous ECHO Autism studies focused on primary care providers (Mazurek et al., 2017). Post-test measures were collected within 2 weeks of the final ECHO session.

### Measures

#### Demographic information and practice patterns

Prior to beginning the pilot, participants completed a survey in which they reported demographic (age, race, ethnicity, sex) and professional (practice setting, specialty, level of education, ASD experience) characteristics. Participants also reported their personal motivation for participating in ECHO Autism as well as any perceived barriers to treating autistic children in their respective practice. This survey was adapted from Mazurek and colleagues (2017).

#### Self-efficacy

Provider self-efficacy was measured at both pre- and post-training via an adapted, shortened version of the Primary Care Autism Self-Efficacy (PCASE) Survey (Mazurek et al., 2017). This measure was originally used for medical providers and was shortened to include only relevant areas for mental health providers. Items were organized into two sub-scales including “co-occurring mental health competencies” and “resource and referral competencies.” Participants responded to each item with their respective level of confidence in effectively enacting the treatment strategy described; levels of confidence were reported on a 1- to 6-point Likert-type scale ranging from a low score of “no confidence” to a high score of “highly confident/expert.” Both total and sub-scales scores were used for analysis.

#### Knowledge test

Provider autism knowledge was assessed at both pre- and post-training via a 20-item Autism Knowledge test that was created by the lead author specifically for this mental health provider population. Items related directly to the content of the ECHO Autism sessions, including knowledge of the prevalence and diagnostic criteria for ASD; strategies to adapt evidence-based treatments, eligibility, and accessibility of school-based ASD services; and mental health co-occurring symptoms and prevalence rates.

#### Problem-solving

To assess changes in provider’s clinical problem-solving skills using evidence-based strategies that were discussed during ECHO Autism sessions, providers were given three clinical scenarios and asked how they would approach each situation. For example, one scenario asked participants how they might tailor CBT for an autistic teenage boy with co-occurring anxiety. Participants were asked to provide open-ended responses to problem-solving vignettes that were scored with a coding rubric on a scale from 0 (“no relation to ECHO Autism training content”) to 2 (“congruent with evidence-based practices discussed during ECHO Autism sessions”) with a score of 1 given for responses that appeared somewhat related to training content but were not specific enough to receive a score of 2. A graduate student coder was trained to 80% reliability with the first author, a psychologist. Both coders were blind to participant condition and all vignettes were randomly assigned; 30% of all responses were double-coded with an 80% interrater reliability.

#### Satisfaction

Provider satisfaction was assessed post-training via a 10-item survey in which participants responded to each item on a 1- to 5-point Likert-type scale ranging from “strongly agree” to “strongly disagree” (Mazurek et al., 2017), with 1 indicating the highest level of satisfaction. Participants were also invited to share their thoughts and suggestions for improvement in free-response sections.

### Data analysis plan

Descriptive statistics were collected for all variables including demographic characteristics and assessment measures. We conducted paired-samples *t*-tests to identify significant mean differences between pre- and post-training outcomes at the *p* < 0.05 level. Because some variables were (as expected) non-normally distributed at either pre- or post-test (e.g. ASD knowledge test scores skewed heavily toward the right at post-test), we also conducted non-parametric Wilcoxon signed-rank tests to further confirm results. Results were consistent across both parametric and non-parametric analyses, so only *t*-tests are reported below. To confirm the stability of results, all analyses were run with both 60% and 80% attendance thresholds; because no differences were observed, we chose to report data for all providers with 60% or greater attendance to provide the largest sample size for analysis.

### Community involvement

As described above, community stakeholders including mental health clinicians and caregivers of autistic individuals provided valuable input to guide curriculum development. In addition, a regional parent advocate participated as an expert on the hub team to share a parent perspective with participating providers. Last, this program was conducted as part of a statewide integrated health home community initiative composed of policymakers and state agency leaders dedicated to improving provider capacity for treating individuals with developmental disabilities.

## Results

### Provider and practice characteristics

Practice settings were primarily private group (27.5%) or solo (23.5%) practices. Most of the sample were social workers (37.3%), and approximately 60% had been in practice for more than 6 years. On average, providers reported treating approximately 112.7 children and adolescents per year (*SD* = 146.6), Notably, the number of autistic children and adolescents that providers reported treating was significantly lower (*M* = 20.4, *SD* = 32.5), with 49% of participants having seen fewer than 10 autistic children and adolescents over the last year. Complete provider demographic and practice characteristics are presented in Table 1.

The most frequently endorsed reason for participating in Project ECHO Autism was a “desire to be more comfortable with providing treatment for the complex behavioral and mental health comorbid conditions that can be associated with autism” (88.2%), and the most frequently endorsed barrier to treating autistic children was a “lack of access to autism specialists” (56.9%). Provider interests and barrier responses are presented in full in Table 3.

**Table 3.** Reasons for interest and perceived barriers.

	*n*	%
Reasons for interest in ECHO Autism		
Desire to be more comfortable with providing treatment for behavioral and mental health comorbid conditions	45	88.2
Desire to learn more about autism	41	80.4
Increased access to specialists for your patients	31	60.8
Continuing education credits	20	39.2
Increased networking with colleagues	17	33.3
Perceived barriers to providing treatment		
Lack of access to autism specialists	29	56.9
Lack of confidence in my ability to manage mental health problems in children with autism	26	51.0
Lack of confidence in my ability to manage behavioral issues in children with autism	24	47.1
Lack of knowledge about autism resources	24	47.1
Lack of prior training in autism	23	45.1
Lack of time	13	25.5
Lack of knowledge about autism symptoms	10	19.6
Lack of support from administration	6	11.8
Inadequate reimbursement	5	9.8

ECHO: Extension for Community Healthcare Outcomes.

### Self-efficacy, knowledge, and problem-solving

**Figure 1.**
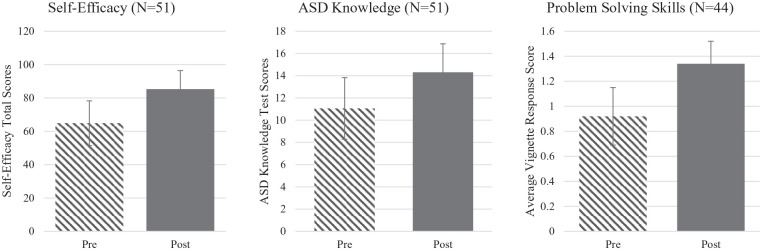
Mean changes in provider self-efficacy, ASD knowledge, and problem-solving skills. *Note*: All differences = *p* < 0.001.

Paired-samples *t*-tests were conducted to compare providers’ responses before and after participation in ECHO (see Figure 1). At post-test, mental health providers collectively scored higher on the Autism Knowledge test (*M* = 14.31, *SD* = 2.56) than at pre-test (*M* = 11.06, *SD* = 2.77), demonstrating significant improvement in their knowledge of ASD prevalence and symptomatology (*t*(51) = −7.24, *p* < 0.001). Similarly, significant increases were observed in provider self-efficacy per report on the adapted PCASE at post-test (*M* = 85.29, *SD* = 11.10) compared to pre-test (*M* = 64.90, *SD* = 13.36) across all items (*t*(51) = −14.20, *p* < 0.001). Specifically, providers indicated a substantial rise in confidence in co-occurring mental health competencies from pre-test (*M* = 40.75, *SD* = 8.91) to post-test (*M* = 55.06, *SD* = 7.41); *t*(51) = −14.44, *p* < 0.001), as well as additional resource and referral competencies from pre-test (*M* = 24.14, *SD* = 5.73) to post-test (*M* = 30.16, *SD* = 4.53); *t*(51) = −9.76, *p* < 0.001). Self-efficacy was reported on a scale of 1 (“no confidence”) to 6 (“highly confident/expert”); significant increases in mean scores from pre-test (*M* = 3.24, *SD* = 0.66) to post-test (*M* = 4.26, *SD* = 0.55; *t*(51) = −14.35, *p* < 0.001) were also observed.

Finally, the subset of mental health providers who provided written responses to vignettes (*n* = 44) was examined. The remaining seven providers did not complete the open-ended vignette questions. Providers demonstrated significant increases in their clinical problem-solving abilities across each vignette with an average change from pre-test (*M* = 0.92, *SD* = 0.23) to post-test (*M* = 1.34, *SD* = 0.18); *t*(43) = −6.32, *p* < 0.001), suggesting an increased awareness in best-practice treatment considerations for autistic individuals. Prior to participation in ECHO Autism, providers generally responded with typical CBT practice recommendations without adaptations to fit autistic clients (e.g. “provide psychoeducation on the relationship between thoughts, feelings, and behaviors” or “challenge cognitive distortions”). After participation in ECHO Autism sessions, provider responses included more specific evidence-based strategies for autistic clients (e.g. “use visual supports to teach coping skills”).

### Provider satisfaction

At post-test, mental health providers reported high satisfaction with their experience participating in this ECHO Autism pilot (*M* = 1.32, range = 1–2). Responses were measured on a 5-point scale, with “1” indicating the highest degree of satisfaction. Participants additionally had the opportunity to provide qualitative feedback on their experience, and responses were likewise highly positive. Across cohorts, providers reported that their skills and knowledge had grown significantly; multiple providers praised the experience as one of the “best trainings” they had attended. Responses included several suggestions to make such training “mandatory” for all mental health professionals. In addition, providers identified both the didactic and case presentation portions of each ECHO session as engaging and highly informative. Several providers noted appreciation of the opportunity to learn from and network with an interdisciplinary hub team. Suggestions for improvement included ideas for additional didactic topics (e.g. gender and ASD) and recommendations for future ECHO Autism cohorts made up of previous participants to gain advanced-level training including additional opportunities for case presentations and feedback as participants continue to implement the evidence-based practices that they learned.

## Discussion

Despite the high prevalence of co-occurring ASD and mental health conditions (Lai et al., 2019) and the establishment of evidence-based adapted mental health treatments for ASD (Steinbrenner et al., 2020; Walters et al., 2016), there is a significant lack of treatment available for those who need it. Autistic individuals diagnosed with co-occurring mental health conditions often face hurdles while navigating service delivery systems, as care coordination is fragmented between mental health and developmental disability service agencies (Maddox & Gaus, 2019). Beyond these macro-system silos, community mental health specialists report co-occurring ASD as a challenge for which they have not been trained (Brookman-Frazee, Drahota, et al., 2012), leading to low levels of ASD knowledge and provider self-efficacy (Maddox, Crabbe, Beidas, et al., 2019), though motivation to engage in ASD-specific training is high (Brookman-Frazee, Drahota, et al., 2012).

Preliminary data from this pilot study suggest that Project ECHO Autism may be a feasible tele-mentoring approach to disseminate ASD expertise to community mental health providers. During initial recruitment, demand was significant; many providers were waitlisted for future ECHO clinics due to the capacity of this first pilot. This level of interest supports previous research suggesting that mental health providers are motivated to learn how to provide evidence-based adapted care to autistic clients (Brookman-Frazee, Drahota, et al., 2012). Indeed, attendance was high, with the majority (88.2%) of providers included in this study attending at least 80% of ECHO sessions.

Participating providers were diverse in their professional experience, reporting highly variable years in practice and number of autistic clients seen each year per practice. Approximately 51% of participants denied having ever received prior ASD-specific training. The most frequent reason for participating in ECHO Autism was a desire to feel more comfortable providing mental health treatment for autistic individuals, followed closely by the desire to learn more about autism. Unsurprisingly, the most frequently endorsed barrier to providing this treatment was the very obstacle this pilot study aimed to ameliorate: a lack of access to autism specialists. Barriers including a lack of confidence in managing behavioral and mental health problems for autistic children were also highly endorsed in this group. After utilizing the ECHO Autism model to move ASD expertise from specialists to community mental health providers, we observed significant gains across all measures in provider self-efficacy, knowledge, and problem-solving.

Providers demonstrated significantly higher levels of confidence in treating co-occurring ASD and mental health conditions post-training, specifically reporting greater self-efficacy in their ability to (1) assess and treat ADHD, anxiety, and challenging behavior for autistic children and adolescents; (2) provide adapted CBT and behavioral treatment; (3) guide parents in the use of behavioral strategies for challenging and self-injurious behaviors; and (4) utilize visual supports, emotion regulation, social competency, and functional communication strategies in treatment. Similarly, providers scored significantly higher in the ASD Knowledge Survey at post-test, suggesting improved competence in understanding autism prevalence, symptomatology, service acquisition, and evidence-based treatment options adapted for autistic clients. The subset of participants who provided responses to vignettes likewise demonstrated a significant increase in problem-solving skills after completion of the pilot study, identifying more ASD-specific evidence-based strategies to address mental health concerns in written case conceptualizations at post-test. Moreover, mental health providers reported high satisfaction with the program. Taken together, results suggest that Project ECHO Autism may be an effective training method to empower community mental health providers to deliver evidence-based treatment to autistic individuals with co-occurring mental health conditions.

In addition to its efficacy, the accessibility afforded to rural providers through this tele-mentoring and consultation platform greatly enhances the feasibility of ECHO Autism as a dissemination model. Barriers to mental healthcare in rural communities are exacerbated by provider shortages and a lack of supervision and professional development opportunities available for those in practice (e.g. consultation with colleagues, specialized trainings) (Smalley et al., 2010). While previous ASD-focused trainings for community mental health providers have demonstrated significant increases in provider competence, the opportunity may often be eclipsed by limited capacity and travel costs. By offering didactic presentations and case consultation virtually, the ECHO hub team of ASD experts reached providers in 16 different rural counties (45.1% of the sample) that may not have been able to access training otherwise. This unique combination of efficacy and accessibility preserves the valuable interactive elements of professional development opportunities while mitigating barriers to attending.

These results have important implications for service access and treatment provision for affected families. As provider self-efficacy and ASD-specific knowledge increase, we anticipate a parallel increase in access to evidence-based services for autistic individuals with co-occurring mental health conditions. Given the substantial burden families face while navigating services for their autistic children (including long waitlists, insurance difficulties, and eligibility restrictions), this sort of training has the potential to mitigate gaps in service access and get tailored treatment into the hands of those who need it sooner.

### Limitations and future directions

While these preliminary findings were encouraging and included a group of participants with diverse backgrounds and from rural counties, a large-scale randomized controlled trial with a more racially and ethnically diverse participant group is needed to fully evaluate the Project ECHO Autism Mental Health program. It is likewise important to note that to preserve the interactive engagement of didactic sessions, each cohort was purposefully limited to a maximum capacity of 25. Future work with larger samples sizes is needed to document the generalizability of study findings.

This pilot study was also limited in its lack of a control or comparison group and reliance on self-report measures and thus may be impacted by social desirability on the part of participating clinicians. To minimize the influence of social desirability and to include a more objective measure of provider outcomes, pre- and post-vignette responses were utilized and coded. These results paralleled the self-reports from providers, offering encouraging evidence for the implementation of more carefully controlled larger research trials. In addition, while information on prior autism-specific training was collected and analyzed, further information on providers’ prior use of evidence-based practice would be helpful to conceptualize gains. Ideally, changes in provider knowledge and self-efficacy will lead to increased access to care and increased use of evidence-based intervention practices by providers. In this pilot study, we were not able to directly measure whether an increase in autistic clients among providers’ caseloads occurred. Research is also needed to document the impact of provider training on practice changes and client outcomes and whether provider perception of barriers to treating autistic clients changed after their participation. As the ECHO Autism platform continues to expand in application, further examination of the psychometric properties of these measures (e.g. self-efficacy and autism knowledge questionnaires) will become increasingly important. In addition, future research is needed on the maintenance of provider gains. We plan to collect follow-up measures from this cohort of providers to assess for sustained gains.

Finally, it may also be beneficial to consider grouping cohorts by the developmental age of each provider’s respective patient pool to enable more focused intervention dissemination in didactic sessions. Notably, this Project ECHO Autism Mental Health pilot received substantial interest from interventionists from our state early intervention program. Broadening the scope of participating providers to include cohorts grouped by age (e.g. early intervention, school counselors, adult therapists) could play a significant role in expanding access for rural families.

## Conclusion

While barriers to service access are steep for families of autistic children with co-occurring mental health conditions, mental health provider motivation to meet those needs is likewise high. Project ECHO Autism is one feasible solution to this gap between provider knowledge and service need and has demonstrated significant gains in provider knowledge, self-efficacy, and problem-solving skills in this mental health–focused pilot. These gains may increase accessibility of services for autistic individuals such that families seeking treatment for co-occurring challenges can find appropriate evidence-based care within their own communities more quickly rather than being referred out to specialists who often have long waitlists and require long drives, particularly for rural families.
